# Sixty years trying to define the malaria burden in Africa: have we made any progress?

**DOI:** 10.1186/s12916-014-0227-x

**Published:** 2014-12-12

**Authors:** Robert W Snow

**Affiliations:** Spatial Health Metrics Group, Department of Public Health Research, KEMRI-Welcome Trust Research Program, Nairobi, Kenya; Centre for Tropical Medicine & Global Health, Nuffield Department of Clinical Medicine, University of Oxford, CCVTM, Oxford, UK

**Keywords:** Malaria mortality, Morbidity, Parasitemia, Measurement, Monitoring, Africa

## Abstract

Controversy surrounds the precise numbers of malaria deaths and clinical episodes in Africa. This would not have surprised malariologists working in Africa 60 years ago as they began to unravel the enigma that is ‘malaria’. Malaria is a complex disease manifesting as a multitude of symptoms, degrees of severity and indirect morbid consequences. Clinical immunity develops quickly and the presence of infection cannot always be used to distinguish between malaria and other illnesses. During the 1950s and 1960s parasite prevalence was used in preference to statistics on malaria mortality and morbidity. An argument is made for a resurrection of this measure of the quantity of malaria across Africa as a more reliable means to understand the impact of control.

## The first estimates of *Plasmodium falciparum* mortality in Africa

During a visit to Uganda and Kenya in 1929, SP James wrote ‘*… there is, as yet, little or no knowledge even of the symptoms and effects of the chronic infestations with malaria parasites from which many native children and adults suffer, to say nothing of the wide gaps in knowledge of* [sic] *regarding the distribution, relative incidence and seasonal prevalence of different species of the malaria parasite*’ [1].

Over the next 30 years, epidemiological studies improved our basic understanding of the clinical impact of malaria infection among communities living under stable, endemic settings in Nigeria [2-4], Liberia [5], Ghana [6,7], the Belgian Congo [8,9], Kenya [10,11] and Tanzania [12,13]. All these studies identified the power and rapidity of natural, repeat parasite exposure on the development of clinical immunity, the resultant absence of disease in adults and how immunity confounded any reliable estimations of the clinical burden posed by infection in rural communities in Africa.

In his comprehensive treatise on malaria, WF Boyd stated that ‘…*there is little value to reports received from physicians .... malaria morbidity reporting, even where malaria is classed as a reportable disease, is notoriously inadequate and unreliable, and nowhere as far as is known to us personally, is it sufficiently complete to be put to epidemiological use*’ [14]. According to those writing on their experiences, the consensus was that ‘*There is little doubt that the diagnosis of malaria in the immune or semi-immune African is subject as often as not to individual interpretation of clinical symptoms by the medical officer...... to complicate the issue, a positive blood-slide from the indigenous inhabitant of West Africa is not necessarily a criterion of the diagnosis*’ [4].

Nevertheless, there was pressure to articulate the malaria burden in Africa as part of the World Health Organization’s Global Malaria Eradication Programme (GMEP). A review of autopsy findings in Lagos performed on 3,085 children younger than 15-years old between 1933 and 1948 [4], formed the basis upon which it was estimated that 5% of children born in Africa would die before their fifth birthday from malaria and, by extrapolation, led to approximately 1 million malaria deaths every year. The ‘million malaria deaths’ became an accepted prelude to any report on malaria in Africa for decades.

### Renewed interest in clinical epidemiology of malaria and new numbers: 1990s

It took another forty years before an interest in the clinical epidemiology of *Plasmodium falciparum,* the most significant malaria parasite transmitted in Africa in terms of disease burden, was to re-emerge [15]. Studies using active surveillance for fever with infection, hospital-based detection of the phenotypes of severe malaria and community-based mortality surveillance increased across Africa generating a wealth of new information. New burden estimates began to emerge based on replications of single studies such as those done by Bruce-Chwatt in the 1950s [16,17].

Toward the end of the 1990s, Africa was in the grips of a malaria epidemic [18]. The need to raise the profile of malaria in Africa, as part of advocacy for an increase in financing for the nascent Roll Back Malaria initiative [19], demanded new numbers. At the same time novel approaches to estimating global cause-specific mortality and disability burdens were gaining credibility for setting international health priorities [20]. In 1999, combinations of data were used to provide a new estimate of the malaria mortality burden in Africa based on assemblies of survey data on clinical attack rates and verbal autopsy or confirmed malaria death rates [21]. This analysis estimated there were more than 221 million febrile clinical events due to *P. falciparum*. Approximately one million deaths were directly attributable to malaria in 1995 [21], a similar finding to that of Bruce-Chwatt’s nearly 50 years earlier. Since then adaptations of this approach have been championed by many (including the author), providing new, refined numbers through increasingly complex models and marginal increases in empirical epidemiological data [22-41].

Generating new numbers is at risk of becoming an industry, no longer informed by adequate biological, clinical and public health reflection or need. Do we really know how many clinical events or deaths are due to *P. falciparum* in Africa? More importantly, are we measuring a quantity that is malaria morbidity and mortality?

### Why is it so difficult to measure the malaria burden in Africa?

Even with comparatively sophisticated surveillance, morbid and fatal events due to malaria in Africa are often treated outside formal health systems and cause of death largely unverifiable. Malaria is a complex disease manifesting as a multitude of symptoms, degrees of severity, with indirect morbid consequences. Without extensive laboratory testing, most symptoms of malaria are indistinguishable from other major causes of morbidity and mortality in young children. Clinical immunity develops quickly during early childhood following repeated parasite exposure, yet how many new infection encounters result in premunition against disease and why some children die and others survive remains unclear [15]. Because immunity develops throughout childhood, the presence of infection cannot always be used to distinguish between malaria and other illnesses sharing similar presenting symptoms.

Recent disease burden estimations have focused almost entirely on direct (those due to the defined clinical syndromes of malaria) measures of morbidity and mortality. For mortality the survey approaches assume that one can use a history from a bereaved relative, a verbal autopsy, to unambiguously assign a death to a single cause, malaria. This is a challenge for even the most experienced clinicians with a severely ill patient in front of them and supported by the best possible laboratory and diagnostic services. Not surprisingly, comparisons of verbal autopsy diagnoses made weeks or months after a child had died of confirmed malaria in hospital have all shown poor sensitivity across Africa [42]. Some have argued that, despite all its limitations, the verbal autopsy is the best tool we currently have [43], but it may be too blunt to provide any reliable data on deaths that are an immediate consequence of a single malaria episode.

Epidemiological models of morbidity do not recognize the intrinsic difficulties of measuring these events. Case definitions change with age and with endemicity [44-50], passive versus active case detection provides very different clinical endpoints [51] and repeat weekly examinations of the same individual alter the ‘natural’ incidence of disease [52]. Standardized clinical endpoints during randomized control trials of new interventions provide important efficacy data, but do not reflect a natural disease burden within trial sites or between sites across different transmission conditions in Africa.

Theory suggests that the association between malaria mortality and transmission intensity is non-linear [53]; the few data available on severe, life-threatening disease support the position of a non-linear association with transmission intensity that saturates at moderate levels of repeat parasite exposure [54,55]. In reality, without better measures of disease outcome, the precise relationship between mortality and malaria transmission remains unknown [56].

As part of recent efforts to define the *P. falciparum* global burden 25 estimates of epidemiologically measured incidence from 16 locations between 1993 and 2001 were used to interpolate risks across the African continent [30]; for mortality, results from 110 verbal autopsy studies from 64 locations and 11 ‘national’ studies in nine African countries were used to provide estimates by year by country between 1980 and 2013 [37,41]. The paucity of empirical disease data in time and across the vast space occupied by diverse epidemiological, social and health system conditions is striking, providing a constant barrier to the generalizability of this approach to burden estimation.

Providing modelled uncertainty around point estimates of morbidity and mortality aims to provide statistical legitimacy for the interpolated burden numbers. The width of the confidence intervals either side of the median point estimates are often numerically the same as the median estimate. Inevitably, the confidence intervals overlap between different modelled estimates, leading some to argue that newer modelling approaches are as ‘good’ as older modelling approaches [57]. A more honest interpretation is that we cannot be statistically confident in any modelled estimate of malaria morbidity or mortality in Africa.

There continues to be a failure to triangulate model predictions with other information on disease burden in Africa. The most recent malaria incidence and mortality models propose that there were more than 19,000 malaria cases and 164 malaria deaths in 2013 in Swaziland [41]. During a national sample survey of infection in 2010 only two infections (one *P. falciparum* and one *P. malariae*) were identified by polymerase chain reaction [58]; malaria is a notifiable disease and case investigations of 779 malaria events showed that only 404 cases over three years (2010 to 2012) were a result of locally acquired infections [59]. The Rwandan government, aiming for elimination, might be equally surprised by the estimated 3,569 deaths directly attributed to *P. falciparum* in 2013 [41]. These examples highlight a more general point: we need to evolve a culture of careful multiple data investigation, triangulation and interrogation as a check on modelling approaches to disease burden at global scales.

### Indirect effects of parasitization

The standard global disease burden approach assumes that one child dies from one disease [20]; as such, significant indirect consequences of malaria infection are ignored. The consequences of reducing malaria parasite exposure on non-malaria mortality have been described during aggressive vector control across Africa. At Pare-Taveta, on the Kenyan-Tanzanian border, three years of house spraying reduced parasite rates from 60% to 5% and was associated with a halving of infant and under-five mortality that reverted to pre-intervention levels after spraying ceased [60,61]. Indoor house spraying with residual insecticides in a high transmission area of Kenya resulted in an almost immediate 96% reduction in parasite exposure that led within three years to a 44% reduction in crude death rates among all age groups and a 40% reduction in infant mortality [62]. Recently, eight rounds of house-spraying and distribution of insecticide treated nets on the island of Bioko, Equatorial Guinea, between 2004 and 2008 resulted in 95% effective coverage of children younger than five-years old, reduced infection prevalence from 42% to 18% and resulted in a reduction in under-five all-cause mortality from 157 to 55 per 1,000 live births [63].

The immediacy of the impact, the effect size and evidence of rebound following cessation of control, all suggest that the reductions seen in overall mortality must be explained by the reductions in parasite exposure and that these reductions are greater than can be explained through a reduction in malaria-specific mortality alone.

The indirect consequences of malaria infection may be mediated through a number of mechanisms [64]. These include the insidious effects of chronic or repeated infection that lead to increased risks of severe anemia in pregnant women and children, growth retardation of fetuses and low-birth weight newborns [27,65,66]. There is also compelling evidence of the role played by *P. falciparum* infection in increasing the risks of invasive bacterial disease, most notably non-typhoidal Salmonellae [67-69]. When malaria transmission dramatically declined over ten years on the Kenyan coast the incidence of life-threatening gram-negative bacteremia was halved [70], confirming that this was directly related to malaria transmission. Further, there appears to be a protective effect of sickle cell trait against invasive bacterial disease, of the same order as its protection against malaria [70].

### What can we measure?

The indirect impact of malaria infection may exceed any direct mortality outcomes, even if the latter could be measured. All-cause mortality is a definable endpoint and it has been argued that one should view malaria infection as a risk factor for survival, in similar ways to how under nutrition is treated as a risk [71]. This is important because it leads us onto what malaria quantity can be measured.

The complex direct and indirect public health burden posed by malaria in Africa is predominantly caused by a single pathogen, *P. falciparum*. Other high burden diseases are usually measured from routine survey data on the prevalence and incidence of the pathogen. Unlike HIV, the direct relationship between malaria infection and disease outcomes is complicated by genetically regulated, naturally acquired exposure-dependent immunity and less well understood variations in parasite virulence. As with all diagnostics for most pathogens, there are margins of error in being able to detect malaria parasites in the peripheral blood of infected hosts [72]. But infection can be measured with a much higher degree of certainty, more rapidly and more ubiquitously than the symptomatic consequences of infection based on a constellation of common childhood symptoms.

In Boyd’s manual on malariology it states that ‘*It is inexcusable to initiate control activities in any community without a prior survey to determine … the endemic level at which malaria is prevailing, and the extent of its localization…* ‘ [13]. During the GMEP era the prevalence of the malaria parasite in a community was viewed as a reliable index of risk that would define how interventions were spatially targeted, and progress measured [73-75]. National surveys were undertaken in thousands of villages across Africa as part of ‘pre-eradication’ reconnaissance. During experimental stages of elimination, parasite ‘incidence’ among infants born during periods of intervention was used to measure the immediacy of control impact [76].

There has been a renaissance in measuring infection prevalence during national sample household malaria indicator surveys since 2005. These more recent national surveys are poor substitutes for those designed and undertaken during the 1950s and 1960s. Current national malaria parasite prevalence surveys often cover fewer than 20 children younger than five years of age per sampled cluster and rarely consider infection prevalence among the entire community or provide information on multiple parasite or life-cycle stages of detected infections. Nevertheless, when combined with other available survey data, notably the revival of school-based measures of malaria risk [77], these data provide a unique opportunity to interpolate infection risks across space and time. The ubiquity and comparative sensitivity of parasite prevalence as a metric of risk has made it possible, for the first time, to measure changes in parasite exposure across Africa based on 26,746 surveys at 21,341 locations [78].

It has long been recognized that when transmission becomes less intense and unstable, there is a closer association between infection prevalence and disease. Disease is theoretically easier to measure without the confounding influence of immunity [79]. Conversely, under conditions of regular parasite exposure disease is much harder to define and the amount of malaria in a community is better measured by the prevalence of the parasite during a cross-sectional survey [79]. The level of transmission one needs to consider when measuring disease or infection is not clear [80]; however, if one assumes that when prevalence is more than 5% disease is hard to measure, then this currently covers 519 million people living in 41 countries of sub-Saharan Africa [78].

## Conclusions

Are we any less ignorant of malaria’s role in the health of communities in Africa since SP James visited East Africa in 1929? We know much more now on the protective role of acquired immunity, the clinical presentations and pathology of malaria as a disease, how disease phenotypes and age-patterns vary according to the intensity of transmission, the diversity of parasite exposure across the continent and the indirect consequences of malaria infection on health.

Multilateral agencies continue to demand actual numbers of a disease intrinsically hard to define, tending to focus on the point median estimates and ignoring the confidence interval. People often argue that some numbers are better than none. Sixty years ago this was probably a fair position; 15 to 20 years ago this might have been legitimate at a time when a case needed to be made to highlight the 40 year neglect of malaria and its threat to global development. At the launch of the GMEP and the more recent Roll Back Malaria (RBM) initiatives, numbers have been useful advocacy tools.

In the absence of reliable civil and health registration systems across a large part of stable endemic sub-Saharan Africa there are two things we can measure: whether someone has died (Millennium Development Goal 4) and whether someone is infected with the malaria parasite (not included in any development goals). The former encompasses the complex array of all risks posed by infection with malaria parasites; the latter is the etiological risk factor for premature mortality. Both are measurable with an acceptable degree of certainty. Yet it remains impossible to provide a single figure of the number of deaths or clinical events resulting from *P. falciparum* infection; or more importantly, how many of these events have been averted since the launch of unprecedented overseas development assistance since 2000.

It is a tragedy that, despite billions of health dollars spent on the control of malaria in Africa since 2000, we remain so uncertain about the health impact of this funding in 2014. Among countries at the margins of stable transmission in Africa, malaria elimination is now viewed, once again, as possible. In these areas, most infections will lead to an important disease outcome unaffected by acquired immunity and countries will require a sophisticated system of disease surveillance to track progress toward the ambitious target of no new clinical events. However, the majority of Africa’s public health burden caused by *P. falciparum* does not occur at the margins but in the middle belt of sub-Saharan Africa where intense transmission leads to immunity within the first few years of life and the unique distinction of a malaria disease event is much harder to define. Investing now in longitudinal, more traditional, epidemiological metrics of malaria including species-specific infection prevalence, mosquito vector species abundance, bionomics and infectivity, accompanied by carefully assembled, quality assured clinical data from hospitals will provide public health specialists an opportunity to examine the success of continued investment in current control tools. Relying upon models based on inadequate, incomplete data of poorly defined disease outcomes should no longer be acceptable.
